# Correlation of serum Meteorin-like (Metrnl) level with type 2 diabetic peripheral neuropathy

**DOI:** 10.1186/s12902-024-01616-2

**Published:** 2024-06-07

**Authors:** Caixia Yao, Hongman Zhang, Li Wang, Jianbo Li

**Affiliations:** 1 Department of Endocrinology and Metabolism, the First Affiliated Hospital of Nanjing Medical University, Nanjing, Jiangsu China; 2 Department of Endocrinology and Metabolism, Gaochun Hospital Affiliated to Jiangsu University, Nanjing, Jiangsu China; 3 Department of Endocrinology, Huai’an First People’s Hospital, Nanjing Medical University, Huai’an, Jiangsu China

**Keywords:** Meteorin-like, Diabetic peripheral neuropathy, Type 2 diabetes mellitus

## Abstract

**Objective:**

Meteorin-like (Metrnl), a secreted myokine, is a newly discovered neurotrophic factor. The aim of this study was to determine if there is a correlation between the Metrnl level and diabetic peripheral neuropathy (DPN).

**Methods:**

The investigation was conducted on a sample of 80 patients with type 2 diabetes mellitus (T2DM) and 60 healthy controls. The T2DM patients were categorized into two subgroups based on skin biopsy: the DPN subgroup (*n* = 20) and the diabetes without neuropathy subgroup (*n* = 60).

**Results:**

The T2DM groups had higher serum Metrnl concentrations compared with the controls. The serum Metrnl concentration was significantly lower in the DPN group than in T2DM patients without neuropathy. Logistic regression analysis demonstrated a notable correlation between serum Metrnl and DPN (OR: 0.997, 95% CI: 0.995–1.000, *P* < 0.05). Serum Metrnl level was negatively correlated with age and SBP after a simple logistic regression analysis.

**Conclusion:**

Serum Metrnl concentration is independently correlated with DPN.

## Introduction

Diabetic peripheral neuropathy (DPN) is a serious complication of diabetes that affects approximately 30–50% of people with diabetes [1]. At present, the mechanism of DPN is not clear, and there is a lack of effective treatments. The development of DPN involves mechanisms such as increased polyol pathway activity, non-enzymatic glycation, and oxidative stress [2–4]. Deficiency in neurotrophic factors is another important cause of DPN [5]. During the development of the nervous system, neuron survival, migration, growth, and differentiation are supported by neurotrophic factors. These secreted factors are also essential for the maintenance and plasticity of the nervous system and may also act as therapeutic agents for neurodegenerative diseases and nerve damage [6].

Meteorin-like (Metrnl) is a secreted protein that is homologous to the neurotrophic factor Meteorin (Metrn). Metrnl is found in various tissues, including nervous system tissue, adipose tissue, stromal cells, mucosal tissue, skin, and activated macrophages [7]. Based on Metrnl expression data obtained from the human BIGE (Body Index of Gene Expression) database, Metrnl shows the highest expression in activated monocytes, with digestive and respiratory mucosal tissue and skin following closely behind [8]. Metrnl, a secreted protein similar to Metrn, NGF (nerve growth factor) and GDNF (glial cell derived neurotrophic factor) acts as a neurotrophic factor that nourishes neurons and plays essential roles in the development, maintenance, and regeneration of neurons [7, 9]. Similar to Metrn, Metrnl functions as a neurotrophic factor to affect the migration of neuroblasts, the growth of neurites, and the survival of spiral ganglion neurons [10].

Based on the above findings, one can assume that the circulating Metrnl level might be associated with diabetic neuropathy. Although some data have been reported on circulating Metrnl levels in diabetes [11–14], there is a lack of data on circulatory Metrnl levels in patients with DPN. In this study, we investigated variations in circulating Metrnl levels among type 2 diabetes mellitus (T2DM) patients with and without DPN and those without T2DM.

## Methods

### Patient selection

From March to October 2022, patients with T2DM were selected as inpatients from Gaochun Hospital Affiliated to Jiangsu University. Healthy control participants were recruited from our hospital physical examination center. A total of 80 patients with T2DM were classified into two groups: the DPN subgroup (*n* = 20) and the diabetes without neuropathy (DM) subgroup (*n* = 60). These subgroups were compared with 60 healthy control subjects matched for age and sex. T2DM was diagnosed according to the 1999 World Health Organization diagnostic criteria and the 2021 American Diabetes Association criteria [15, 16]. Patients who had previous neurologic ailments (e.g., diseases affecting the central nervous system, known peripheral vascular disorders, and hyperplasia or tumors derived from neuroendocrine cells), a body mass index (BMI) greater than 30 kg/m^2^ or less than 18 kg/m^2^, severe renal dysfunction [estimated glomerular filtration rate (GFR) < 45 mL/min], severe liver dysfunction (aspartate or alanine aminotransferase higher than three times the normal level), heart failure (New York Heart Association class III or IV), or cancer or infection within the last three months were excluded. To minimize selection bias, participants were chosen randomly rather than being recruited based on any arbitrary basis (such as DPN symptoms). Informed consent was provided by all participants. Approval for the study was granted by the hospital’s scientific and ethical committees (AF/SQ-19/01.0).

### Measurement of anthropometric characteristics and other biochemical parameters

BMI was calculated by the standard formula [BMI = weight (kg) / height (m^2^)]. The systolic blood pressure (SBP) and diastolic blood pressure (DBP) were measured after 15 min of rest using a mercury sphygmomanometer [17]. Following overnight fasting, blood samples were obtained from all participants through venipuncture. Fasting plasma glucose (FPG), triglycerides (TG), total cholesterol (TC), low density lipoprotein-cholesterol (LDL-C), high density lipoprotein-cholesterol (HDL), alanine aminotransferase (ALT), aspartate aminotransferase (AST), and serum creatinine (Scr) were measured using an automatic biochemistry analyzer (AU5800; Beckman Coulter, America). The estimated glomerular filtration rate (GFR) was calculated as the endogenous creatinine clearance using the Cockcroft equation for males: GFR = [140 – age (years) * body weight (kg)] / [0.818 * Scr (µmol/L)]. The GFR was multiplied by 0.85 for females. HbA1c (Glycated Hemoglobin, Type A1c) was determined using high-performance liquid chromatography (HA-8180; ARKRAY, Japan). The urinary albumin concentration was measured by immunophelometry (AU5800; Beckman Coulter, America). The urinary albumin-to-creatinine ratio (ACR) was determined as ACR = urine albumin (mg) / urine creatinine (g). All measurements were repeated twice.

### Enzyme-linked immunosorbent assay (ELISA) determination of serum metrnl level

Samples of human blood were collected and allowed to coagulate for 2 h at room temperature or overnight at 4℃ before centrifugation for approximately 20 min at 1000×g followed by storage at − 80℃ for future use. The serum Metrnl levels were determined using highly sensitive ELISA kits obtained from Amoy Lunchangshuo Biotech, China. Duplicate additions of standards and samples were made to the microtiter plate. The enzyme conjugate was added to the standard hole and the sample hole (excluding the blank hole). After covering the holes with tape, the samples were incubated at 37℃ for 60 min. After washing with washing solution (1X) four times, substrates A and B were added to each well followed by gentle mixing and incubation in the dark at 37 °C for 15 min. The termination remedy was introduced, and the optical density at 450 nm was measured using a microtiter plate reader (VARI0SKAN LUX, Thermo, Germany) within 15 min.

### Neuropathy assessment

**Fig. 1:**
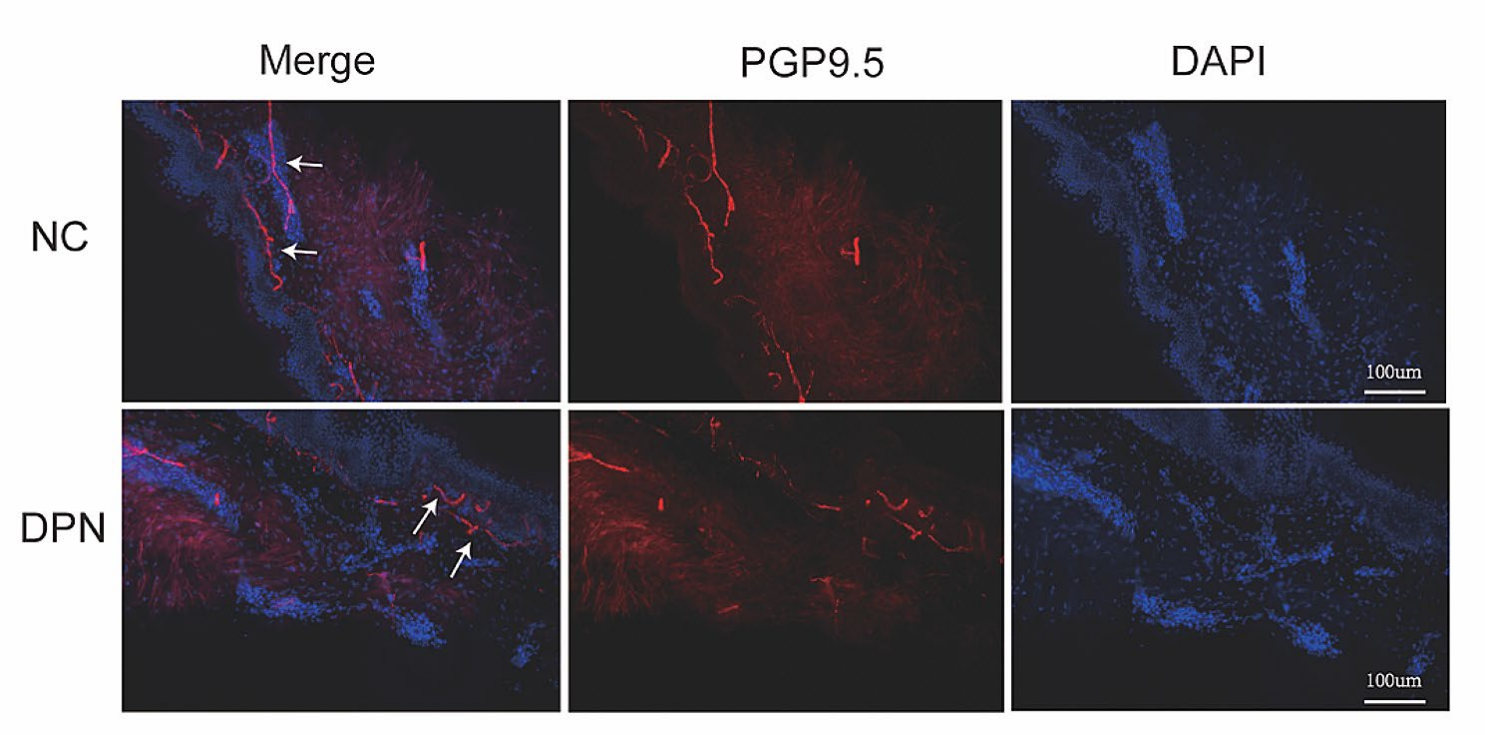
Small nerve fibers in the skin tissue of patients evaluated using PGP9.5 staining. NC, normal control; DPN, diabetic peripheral neuropathy; PGP9.5, protein gene product 9.5. The white arrows show the counted nerve fibers

In our Metabolic Management Center, all participates underwent a thorough assessment based on the neuropathy symptom profile. Neurologic deficits, including unequivocally decreased or absent reflexes and decreased distal sensations for neuropathy, were revealed through physical examination with the following tools based on the modified neuropathy disability score: a 10-g monofilament for touch sensation test (four sites per foot); a pin for pain sensation test; a tendon hammer for reflex test; and a standard 128-Hz tuning fork for vibration sensation test. The indicators were recorded according to the neurological impairment rating: 0 indicating no impairment, 1 indicating a slight impairment, 2 indicating a moderate impairment, 3 indicating a severe impairment, and 4 indicating a complete loss of function or the most severe impairment [18]. Electromyography was used to measure the nerve conduction velocities of ulnar, median, sural, and peroneal nerves on the right side. If two or more nerves exhibited abnormal results, nerve conduction was deemed abnormal [19]. The number of nerve fibers entering the epidermal layer from the dermis through the basement membrane was counted as 1 intraepidermal nerve fiber. The length of the epidermal layer was measured using ImageJ software (Rawak Software Germany). The intraepidermal nerve fiber density (IENFD) in units of number/mm was calculated by dividing the number of nerve fiber numbers in the epidermis by the length of the epidermal layer. The fifth percentile of IENFD in the normal group was the tangent point of the normal value of IENFD; IENFD values below this point were considered to indicate abnormal function of small fiber nerves and diagnosed as DPN (Fig. 1) [20]. All patients with diabetes had no abnormal nerve conduction; skin biopsies in DPN patients showed significant decreases in small nerve fibers with or without mild to moderate deficits in neurological impairment scores.

### Immunohistochemistry and skin biopsy

IENFD was evaluated in DM patients who received a 3-mm punch skin biopsy of the left lateral calf (approximately 10 cm above the lateral malleolus) after local anesthesia (2% lidocaine). The biopsy sample was promptly fixed in 4% paraformaldehyde for 24 h, immersed in 30% sucrose (< 4 h) at 4℃, embedded in optimal cutting temperature-embedding compound, swiftly frozen in liquid nitrogen, and sliced into 50-µm sections utilizing a cryostat (CM1950; Leica, Germany). After washing three times with 0.1 M phosphate buffer for 10 min each time, the floating sections were subjected to a protein block with a 5% bovine serum albumin solution containing 0.3% Triton X-100 at room temperature for 2 h. Subsequently, the sections were incubated overnight in PGP9.5 monoclonal antibody (GB12159, Servicebio, China) at 4 °C. The next day, the samples were washed three times for 10 min each time in 0.1 M phosphate buffer containing 0.05% Tween20 followed by incubation at room temperature in the dark with a secondary antibody for 1.5 h. After rinsing three times for 10 min each time in the dark, the tissues were cover-slipped with an anti-fluorescence quenching medium containing DAPI and examined under a microscope. The number of nerve fibers entering the epidermal layer from the dermis through the basement membrane was considered as one intraepidermal nerve fiber. IENFD was determined in accordance with the established criteria [20, 21].

### Statistical analysis

Frequency and percentage were used to express categorical data, and differences were evaluated by chi-square test. Continuous variables were expressed as the mean and standard deviation. The Kolmogorov–Smirnov test was used to assess the normality of continuous data. Normally distributed data were analyzed by one-way analysis of variance with Bonferroni’s post hoc analysis. Non-normally distributed data were assessed by Kruskal - Wallis test and Bonferroni’s test. Spearman correlation analysis was used to assess the correlations between Metrnl and anthropometric and metabolic parameters. Logarithmic transformation was applied to non-normally distributed data prior to conducting correlation analysis. To eliminate any potential effects of covariates on the serum Metrnl levels, an analysis of covariance was conducted. Furthermore, the correlations between Metrnl and T2DM and DPN were examined by multiple logistic regression. The analyses were conducted using SPSS version 25 (SPSS, USA) with the significance threshold set at *P* < 0.05.

## Results

### Characteristics of the study subjects

There were no obvious differences between the gender makeup and urinary ACR among the three groups. In T2DM patients, the BMI, FBG, HbA1c, TC, LDL-c, TG, SBP, and DBP were increased compared with the healthy controls, whereas HDL-C was decreased (Table 1). Compared to diabetes patients without neuropathy, the diabetes with neuropathy had higher ages and lower GFR (Table 1).

**Table 1:** Clinical characteristics of the subjects

Characteristics	Healthy controls	Diabetes without neuropathy	Diabetes with neuropathy	t/F	*P*
*N*	60	60	20		
Age (years)	52.30 ± 10.13	50.77 ± 13.95	60.60 ± 13.36 ^a, b^	4.845	0.009
Gender (M/F)	32/28	42/18	10/10	4.444	0.108
Duration (years)	—	5.94 ± 6.22	5.76 ± 5.67	0.118	0.907
BMI (kg/m^2^)	22.19 ± 1.40	24.40 ± 3.29 ^a^	23.95 ± 3.10 ^a^	11.265	< 0.001
FBG (mmol/L)	5.28 ± 0.44	12.16 ± 4.31 ^a^	10.70 ± 3.61 ^a^	75.306	< 0.001
HbA1c (%)	5.59 ± 0.31	9.60 ± 2.15 ^a^	8.85 ± 1.80 ^a^	102.463	< 0.001
TC (mmol/L)	4.34 ± 0.59	4.94 ± 1.04 ^a^	5.00 ± 1.80 ^a^	7.071	0.001
TG (mmol/L)	1.14 ± 0.43	2.37 ± 2.52 ^a^	2.63 ± 3.48 ^a^	6.525	0.002
HDL-C (mmol/L)	2.42 ± 1.03	1.20 ± 0.26 ^a^	1.34 ± 0.44 ^a^	46.937	< 0.001
LDL-C (mmol/L)	2.46 ± 0.43	2.95 ± 0.66 ^a^	2.89 ± 0.51 ^a^	12.997	< 0.001
SBP (mmHg)	121.03 ± 7.63	136.22 ± 15.48 ^a^	130.55 ± 18.05 ^a^	20.126	< 0.001
DBP (mmHg)	73.70 ± 4.43	84.98 ± 12.78 ^a^	81.8 ± 9.49 ^a^	21.486	< 0.001
GFR (mL/min)	104.52 ± 6.65	100.51 ± 20.16	83.52 ± 23.34 ^a, b^	12.361	< 0.001
ACR (mg/g)	17.86(10.00, 27.87)	17.14(6.25, 48.87)	20.05(10.09, 32.57)	0.296	0.862
Metrnl (pg/mL)	322.02 ± 191.88	635.08 ± 386.99 ^a^	391.00 ± 210.26 ^b^	17.759	< 0.001

BMI, body mass index; FBG, fasting blood glucose; SBP, systolic blood pressure; DBP, diastolic blood pressure; TG, triglycerides; TC, total cholesterol; HDL-C, high-density lipoprotein cholesterol; LDL-C, low-density lipoprotein cholesterol; ACR, urine albumin to creatinine ratio; GFR, glomerular filtration rate

^a^ Significant versus healthy control subjects

^b^ Significant versus diabetes without neuropathy

### Serum metrnl concentrations

**Fig. 2:**
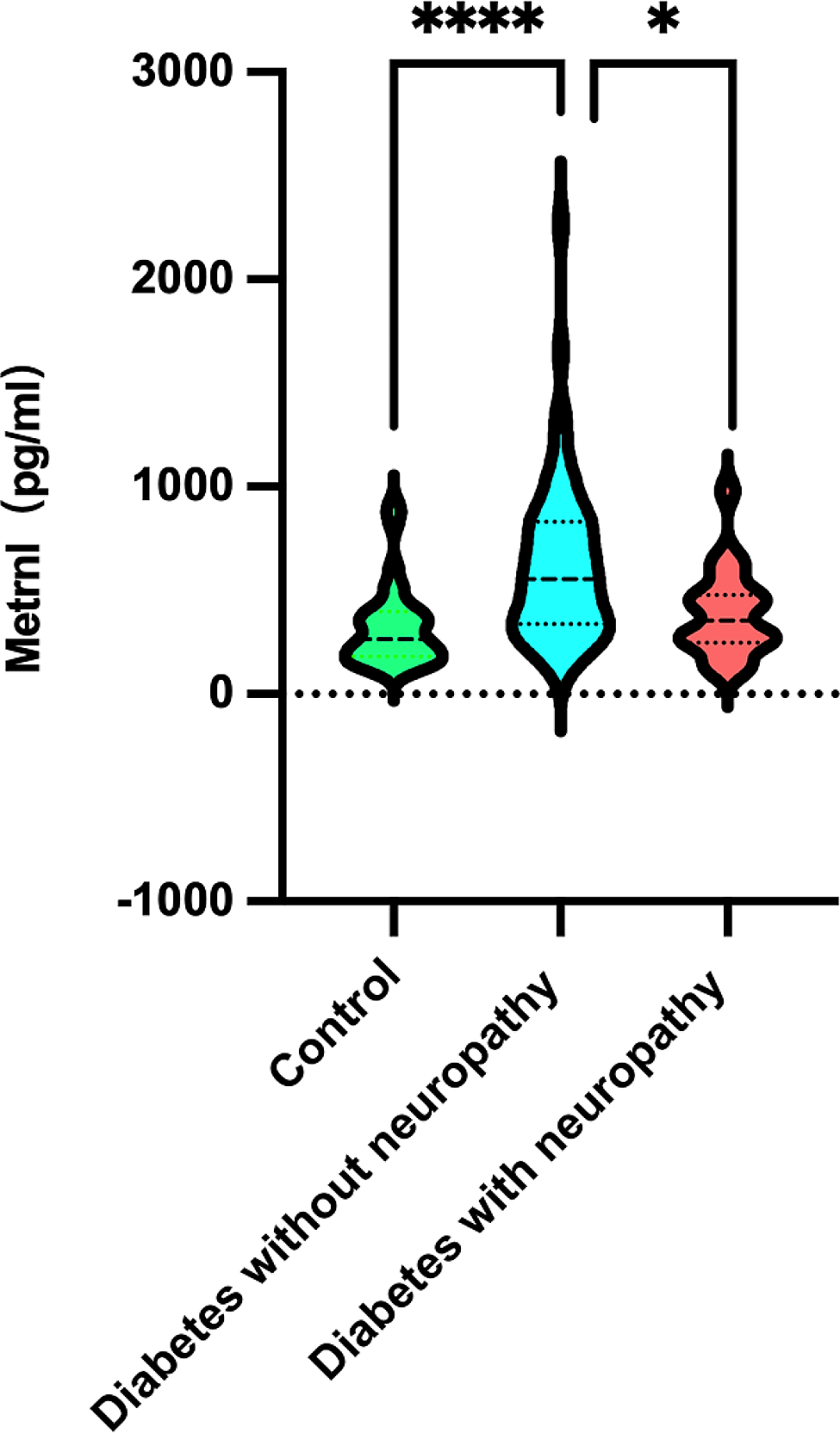
Serum Metrnl levels in T2DM patients and healthy controls. The diabetes without neuropathy subgroup showed significantly increased serum Metrnl concentrations compared with the control group. The serum Metrnl concentrations were significantly lower in the diabetes with neuropathy group compared with the DM group

The diabetes without neuropathy subgroup showed significantly increased serum Metrnl concentrations compared with the control group. Compared to the diabetes without neuropathy, the serum Metrnl level was significantly lower in the diabetes with neuropathy (391.00 ± 210.26 vs. 635.08 ± 386.99, *P* < 0.001; Table 1; Fig. 2).

### Multiple regression analysis of Metrnl concentration and T2DM

T2DM patients exhibited significantly elevated serum Metrnl levels compared to the controls (635.08 ± 386.99 vs. 322.02 ± 191.88, *P* < 0.001). Univariate logistic regression analysis was used to correlate T2DM with body mass index (BMI), lipid profile (TC, HDL-c, LDL-c, and TG), blood pressure (SBP and DBP), GFR, urine ACR, and serum Metrnl. These parameters were then used as input in a multivariate logistic regression model. After conducting multivariate logistic regression analysis, serum Metrnl remained associated with T2DM (Table 2).

**Table 2:** Logistic regression analysis for determining the risk factors for T2DM with or without neuropathy

Characteristics	Simple logistic regression		Multiple logistic regression	
	OR (95% CI)	*P*	OR (95% CI)	*P*
Age (years)	1.006 (0.980–1.033)	0.669	—	—
Gender (M/F)	0.615 (0.310–1.220)	0.164	—	—
BMI (kg/m^2^)	1.457 (1.217–1.745)	< 0.001	1.100 (0.770–1.571)	0.601
TC (mmol/L)	2.296 (1.437–3.668)	0.001	13.347 (1.621–109.894)	0.016
TG (mmol/L)	4.898 (2.429–9.877)	< 0.001	5.052 (0.559–45.662)	0.149
HDL-C (mmol/L)	0.039 (0.012–0.123)	< 0.001	0.004 (0.000–0.087)	< 0.001
LDL-C (mmol/L)	5.113 (2.455–10.647)	< 0.001	2.605 (0.275–24.657)	0.404
SBP (mmHg)	1.101 (1.059–1.146)	< 0.001	1.101 (1.009–1.202)	0.030
DBP (mmHg)	1.166 (1.097–1.239)	< 0.001	1.083 (0.980–1.196)	0.118
GFR (mL/min)	0.970 (0.947–0.993)	0.009	0.905 (0.837–0.978)	0.012
ACR (mg/g)	1.015 (1.000–1.030)	0.046	1.017 (0.981–1.054)	0.357
Metrnl	1.004 (1.002–1.006)	< 0.001	1.004 (1.001–1.008)	0.027

BMI, body mass index; FBG, fasting blood glucose; SBP, systolic blood pressure; DBP, diastolic blood pressure; TG, triglycerides; TC, total cholesterol; HDL-C, high-density lipoprotein cholesterol; LDL-C, low-density lipoprotein cholesterol; ACR, urine albumin to creatinine ratio; GFR, glomerular filtration rate

### Multiple regression analysis of serum metrnl level and DPN

The diabetes patients with neuropathy exhibited lower serum Metrnl concentrations compared to those without neuropathy (391.00 ± 210.26 vs. 635.08 ± 386.99, *P* < 0.001). The correlations between age, GFR, and serum Metrnl with DPN were evaluated by univariate logistic regression analysis. These parameters were then input into a multivariate logistic regression model. Following multivariate logistic regression, the serum Metrnl concentrations remained associated with DPN (Table 3).

**Table 3:** Logistic regression analysis for determining the risk factors of DPN

Characteristics	Simple logistic regression		Multiple logistic regression	
	OR (95% CI)	*P*	OR (95% CI)	*P*
Age (years)	1.056 (1.013–1.102)	0.011	1.012 (0.959–1.068)	0.658
Gender (M/F)	2.333 (0.828–6.575)	0.109	—	—
Duration (years)	0.995 (0.914–1.083)	0.905	—	—
BMI (kg/m^2^)	0.956 (0.813–1.125)	0.591	—	—
FBG (mmol/L)	0.911 (0.795–1.043)	0.177	—	—
HbA1c (%)	0.825 (0.628–1.084)	0.167	—	—
TC (mmol/L)	1.043 (0.673–1.616)	0.851	—	—
TG (mmol/L)	1.032 (0.868–1.225)	0.722	—	—
HDL-C (mmol/L)	3.758 (0.781–18.081)	0.099	—	—
LDL-C (mmol/L)	0.861 (0.379–1.955)	0.721	—	—
SBP (mmHg)	0.977 (0.944–1.011)	0.179	—	—
DBP (mmHg)	0.979 (0.939–1.021)	0.313	—	—
GFR (mL/min)	0.964 (0.939–0.989)	0.005	0.974 (0.942–1.006)	0.110
ACR (mg/g)	0.997 (0.991–1.003)	0.374	—	—
Metrnl	0.997 (0.995–0.999)	0.010	0.997 (0.995–1.000)	0.047

BMI, body mass index; FBG, fasting blood glucose; SBP, systolic blood pressure; DBP, diastolic blood pressure; TG, triglycerides; TC, total cholesterol; HDL-C, high-density lipoprotein cholesterol; LDL-C, low-density lipoprotein cholesterol; ACR, urine albumin to creatinine ratio; GFR, glomerular filtration rate

### Correlation between serum Metrnl and other variables

As shown in Table 4, simple linear regression analysis revealed an inverse correlation between the serum Metrnl level and age in T2DM patients. There is a negative correlation between the serum Metrnl concentrations and SBP in DPN patients (Table 5).

**Table 4:** Correlations between the serum Metrnl concentrations and various parameters in T2DM patients

Characteristics	Simple logistic regression	
	*r*	*P*
Age (years)	**−0.285**	**0.011**
Gender (M/F)	**−**0.070	0.539
Duration (years)	**−**0.087	0.443
BMI (kg/m^2^)	**−**0.026	0.818
FBG (mmol/L)	0.012	0.917
HbA1c(%)	0.016	0.890
TC (mmol/L)	0.205	0.068
TG (mmol/L)	0.099	0.382
HDL-C (mmol/L)	**−**0.043	0.707
LDL-C (mmol/L)	0.160	0.156
SBP (mmHg)	0.156	0.167
DBP (mmHg)	0.127	0.260
GFR (mL/min)	0.201	0.074
ACR (mg/g)	0.127	0.260

BMI, body mass index; FBG, fasting blood glucose; SBP, systolic blood pressure; DBP, diastolic blood pressure; TG, triglycerides; TC, total cholesterol; HDL-C, high-density lipoprotein cholesterol; LDL-C, low-density lipoprotein cholesterol; ACR, urine albumin to creatinine ratio; GFR, glomerular filtration rate

**Table 5:** The correlation between serum Meteorin-like concentrations and various parameters in DPN patients

Characteristics	Simple logistic regression	
	*r*	*P*
Age(years)	-0.326	0.161
Gender(M/F)	-0.074	0.755
Duration(years)	-0.253	0.283
BMI (kg/m2)	-0.018	0.939
FBG(mmol/L)	0.068	0.776
HbA1c	0.068	0.776
TC (mmol/L)	0.282	0.229
TG (mmol/L)	0.174	0.464
HDL-C(mmol/L)	0.150	0.527
LDL-C(mmol/L)	0.334	0.150
SBP (mmHg)	**-0.464**	**0.040**
DBP (mmHg)	-0.398	0.082
GFR (ml/min)	0.333	0.152
ACR (mg/g)	-0.342	0.140

BMI, body mass index; FBG, fasting blood glucose; SBP, systolic blood pressure; DBP, diastolic blood pressure; TG, triglycerides; TC, total cholesterol; HDL-C, high-density lipoprotein cholesterol; LDL-C, low-density lipoprotein cholesterol; ACR, urine albumin to creatinine ratio; GFR, glomerular filtration rate

## Discussion

DPN has a high incidence in T2DM patients and can result in amputation, disability, and potentially death. The pathogenesis of DPN is not yet clear. Many studies have focused on oxidative stress, inflammation, ischemia, and hypoxia caused by long-term exposure to hyperglycemia. We previously found that exogenous neuritin can improve the survival and function of Schwann cells along with the growth of neurons in diabetic rats [5]. This suggests that deficiencies in neurotrophic factors, including the factor Metrnl, may be an important pathogenesis of DPN.

In this study, among T2DM patients, the serum Metrnl concentrations were significantly lower in those with DPN compared with the patients without neuropathy. In addition, the serum Metrnl concentrations were higher in the diabetic patients without neuropathy compared to the healthy controls. Logistic regression analysis revealed an independent correlation between the serum Metrnl level and DPN. Therefore, serum Metrnl may be a novel biomarker for the diagnosis or early recognition of DPN. In the future, Metrnl may serve as a therapeutic agent for DPN.

Previous studies on the relationship between Metrnl and T2DM have not focused on the association between Metrnl and DPN. To our knowledge, this study is the first to examine changes in serum Metrnl levels in patients with DPN.

Some studies have found that serum Metrnl levels are elevated in diabetic patients [11, 13, 22–24], whereas others have indicated decreased Metrnl levels in diabetic patients [12, 14, 25, 26]. In the present study, serum Metrnl levels were elevated in the diabetic group compared to the healthy control group (Fig. 2). Due to the limited number of studies on the relationship between T2DM and serum Metrnl levels, it is difficult to interpret these conflicting findings. The mechanism underlying the association between serum Metrnl level and diabetes is not fully understood. Loffler et al. [27] reported that Metrnl decreased PPAR-α expression, leading to insulin resistance and hyperinsulinemia. Wang et al. [23] reported that high serum Metrnl levels were positively correlated with insulin resistance, BMI, and waist circumference. AlKhairi et al. [13] found that serum Metrnl levels were increased in overweight and obese subjects compared to the health controls. In the current investigation, we found that the BMI was significantly higher in the diabetic groups than in the healthy control group (Table 1). Another explanation for the increased serum Metrnl levels in T2DM patients may be a protective compensatory response to metabolic stress, including Metrnl resistance and insulin resistance [11, 28, 29]. Schmid et al. [26] found that systemic Metrnl was upregulated during massive weight loss. In the guidelines of diabetes societies such as the American Diabetes Association, changes in lifestyle and diet are primarily recommended for the treatment of T2DM. In this study, most patients with diabetes were overweight, and these patients increased their exercise level and experienced weight loss after diagnosis. However, confounding factors such as exercise were not evaluated in the present study.

Wang et al. [14] found that the decrease in serum Metrnl concentration was correlated with diabetic nephropathy. El-Ashmawy et al. [25] found that low serum Metrnl values may be associated with impaired endothelial function and atherosclerosis. Diabetic neuropathy and diabetic nephropathy are both classified as diabetic microangiopathy. In the current study, the serum Metrnl levels were significantly lower in the diabetic neuropathy group compared to the diabetes without neuropathy group. This study demonstrated that among T2DM patients, a significant decrease in the Metrnl level is associated with diabetic neuropathy. The serum Metrnl concentration was found to be independently correlated with DPN based on multiple regression analysis. In addition, the serum Metrnl level was higher in the DPN group than in the healthy control group, although the difference was not statistically significant; this may be due to the small number of DPN samples or the fact that only early small fiber neuropathy occurred in the DPN group. The skin biopsy fluorescent staining did not involve Metrnl because the ELISA results of serum Metrnl were sufficient to support the analytical data; skin biopsy, with a limited tissue specimen size (3-mm diameter per patient), was only used to diagnose DPN. Nevertheless, further investigations are required to validate the potential molecular mechanism of Metrnl in the pathogenesis of DPN.

There are several limitations to this study. First, the study was cross-sectional, making it impossible to establish a causal connection between the serum Metrnl level and T2DM, regardless of the presence of neuropathy. Second, the research focused on prevalent instances of T2DM, potentially overlooking individuals with T2DM who experienced adverse outcomes as a result of severe diabetic complications. Third, it was not possible to entirely dismiss the possibility that bias or other factors (e.g., the duration, intensity, and type of exercise) influenced the results. Finally, the study had a limited sample size; larger studies are needed in the future.

## Conclusion

In summary, serum Metrnl concentration is independently correlated with DPN. Thus, the serum Metrnl concentration could potentially serve as a new biomarker for the early diagnosis and risk assessment of DPN.

## Data Availability

The data that support the findings of this study are available from the corresponding author upon reasonable request. The data are not publicly available due to privacy or ethical restrictions.

## Abbreviations

BMI: Body mass index
FBG: Fasting blood glucose
SBP: Systolic blood pressure
DBP: Diastolic blood pressure
TG: Triglycerides
TC: Total cholesterol
HDL-C: High-density lipoprotein cholesterol
LDL-C: Low-density lipoprotein cholesterol
ACR: Urine albumin to creatinine ratio
GFR: Glomerular filtration rate
